# Real-time Prediction of the Daily Incidence of COVID-19 in 215 Countries and Territories Using Machine Learning: Model Development and Validation

**DOI:** 10.2196/24285

**Published:** 2021-06-14

**Authors:** Yuanyuan Peng, Cuilian Li, Yibiao Rong, Chi Pui Pang, Xinjian Chen, Haoyu Chen

**Affiliations:** 1 School of Electronics and Information Engineering Soochow University Suzhou China; 2 Joint Shantou International Eye Center Shantou University & the Chinese University of Hong Kong Shantou China; 3 College of Engineering Shantou University Shantou China; 4 Department of Ophthalmology and Visual Sciences Chinese University of Hong Kong Hong Kong China (Hong Kong)

**Keywords:** COVID-19, daily incidence, real-time prediction, machine learning, Google Trends, infoveillance, infodemiology, digital health, digital public health, surveillance, prediction, incidence, policy, prevention, model

## Abstract

**Background:**

Advanced prediction of the daily incidence of COVID-19 can aid policy making on the prevention of disease spread, which can profoundly affect people's livelihood. In previous studies, predictions were investigated for single or several countries and territories.

**Objective:**

We aimed to develop models that can be applied for real-time prediction of COVID-19 activity in all individual countries and territories worldwide.

**Methods:**

Data of the previous daily incidence and infoveillance data (search volume data via Google Trends) from 215 individual countries and territories were collected. A random forest regression algorithm was used to train models to predict the daily new confirmed cases 7 days ahead. Several methods were used to optimize the models, including clustering the countries and territories, selecting features according to the importance scores, performing multiple-step forecasting, and upgrading the models at regular intervals. The performance of the models was assessed using the mean absolute error (MAE), root mean square error (RMSE), Pearson correlation coefficient, and Spearman correlation coefficient.

**Results:**

Our models can accurately predict the daily new confirmed cases of COVID-19 in most countries and territories. Of the 215 countries and territories under study, 198 (92.1%) had MAEs <10 and 187 (87.0%) had Pearson correlation coefficients >0.8. For the 215 countries and territories, the mean MAE was 5.42 (range 0.26-15.32), the mean RMSE was 9.27 (range 1.81-24.40), the mean Pearson correlation coefficient was 0.89 (range 0.08-0.99), and the mean Spearman correlation coefficient was 0.84 (range 0.2-1.00).

**Conclusions:**

By integrating previous incidence and Google Trends data, our machine learning algorithm was able to predict the incidence of COVID-19 in most individual countries and territories accurately 7 days ahead.

## Introduction

COVID-19, a highly infectious disease with serious clinical manifestations, was first reported in China in late 2019 and spread to other countries within weeks [1,2]. It was announced as a public health emergency of international concern by the World Health Organization (WHO) on January 30, 2020, and declared a pandemic on March 11, 2020 [3]. As of August 21, 2020, COVID-19 has been reported in 215 countries and territories, with 22,876,009 confirmed cases and 797,289 deaths [4]. The severity of the pandemic is variable in individual countries and territories. In some regions, severe outbreaks, occurred and case numbers took a long time to decrease. In other regions, it took more time for the spread of the disease to affect people. Even in places where the spread has been brought under control, there have been second or third waves of outbreaks, with variable severities of morbidities and mortalities.

Prediction of the incidence of COVID-19 in individual countries and territories is extremely important to provide reference for governments, health care providers, and the general public to prepare management measures to combat the disease. In the early months of 2020, prediction was useful to inform the countries and territories at risk of outbreak to take action for prevention. Currently, although the COVID-19 outbreak has already occurred in almost all regions globally, prediction has merits in monitoring the severity of spreading and recovery and assessing the likelihood of a secondary or tertiary epidemic. COVID-19 may become a seasonal or persistent epidemic in future years, like influenza. Thus, predicting and monitoring COVID-19 activity is needed at present and in the future. As a typical example, Google search data can predict the incidence of influenza effectively [5].

There are reported studies on the prediction of the incidence of COVID-19. A susceptible-exposed-infectious-recovered metapopulation model was used to simulate the epidemic in major cities in China [1]. Our previous study showed that data from internet search engines and social media platforms could predict the incidence of COVID-19 in China [6]. Later, data mining and machine learning techniques were used to predict the incidence of COVID-19 using data from the previous incidence or internet search volumes [7,8]. In these studies, only a few countries were investigated or the world was considered as one region. Furthermore, as the pandemic continues, increasing amounts of data are available for modeling and calibration. The actual situation may be different from previous predictions. For example, Tuli et al [9] predicted that the total number of new cases would reach 97% of the total expected cases on August 14 in the United States; however, actually, there were still tens of thousands of new cases daily. Therefore, an up-to-date and evolving prediction method is needed.

In this study, we aim to develop an efficient and novel methodology for real-time prediction of COVID-19 activity based on the previous daily incidence of COVID-19 and infoveillance data (search volume data via Google Trends) in all individual countries and territories worldwide.

## Methods

### Data Source

Two sets of data were collected. The first data set was the search volume data obtained from the Google Trends service. We collected the Google search volumes of 28 candidate features related to COVID-19 from January 1 to July 26, 2020, in 215 countries and territories. We used 14 terms, including *coronavirus*, *pneumonia*, and *Covid-19*; 6 symptom-related terms [10] (*cough*, *diarrhea*, *fatigue*, *fever*, *nasal congestion*, and *rhinorrhea*); and 5 prevention-related terms (*hand washing*, *hand sanitizer*, *mask*, *social distance*, and *social isolation*. There were two main reasons for selecting these 14 terms for Google Trends data collection: (1) Previous studies [6,10-13] have shown that the internet search data for these terms were correlated with the incidence of COVID-19. (2) These 14 terms are related to COVID-19 in different aspects, such as topics, symptoms, and prevention; therefore, they can reflect its incidence. Two types of Google Trends data were retrieved. The first type was the data of interest over time, defined as the search interest relative to the highest point for the specific term, region, and time interval. Values were calculated on a scale from 0 to 100, where 100 is the peak popularity for the time period. The second type of data, data of interest by region, was retrieved by setting the region to “worldwide” for the given term and time. The values were calculated on a scale from 0 to 100, where 100 is the location with the most popularity as a fraction of total searches in that location. It is notable that we included low search volume regions to obtain data for more regions.

**Figure 1:**
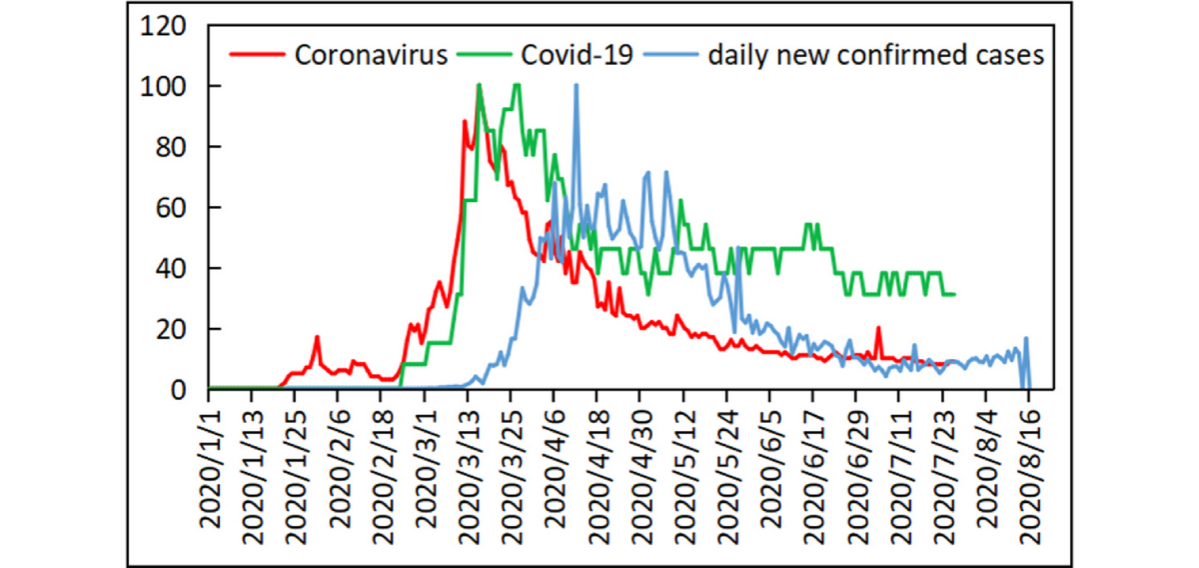
The Google Trends search volumes for the topics *coronavirus* (red line) and *Covid-19* (green line) and the number of daily new confirmed cases (blue line) in the United Kingdom. All values are scaled from 1 to 100.

The second data set was the daily number of new COVID-19 cases from January 10 to August 16, 2020, in 215 countries and territories, obtained from the WHO website [14]. For consistency, the values of the daily number of new COVID-19 cases in the 215 countries and territories were normalized to a scale from 0 to 100. An example showing the search volumes of the terms *coronavirus* and *Covid-19* and the daily incidence of COVID-19 in the United Kingdom is shown in Figure 1.

### Correlation Analysis and Feature Engineering

We conducted two types of correlation analyses in each country or territory to find the right combination of input features. The first analysis was the Spearman correlation between the Google volume data of each feature and daily new confirmed cases with n days of lag (n=7, 14, 21, 28). We obtained the average and maximum Spearman correlation coefficients for different lag days between each Google volume data feature and daily newly confirmed cases in the 215 countries and territories (Multimedia Appendix 1). Each Google volume data feature was related to the daily new confirmed cases in the 215 countries and territories. The top related item was the interest over time in *coronavirus*. We found a better correlation with the 21-day lag than with the other days of lag. Therefore, we combined 28 Google volume data features with daily new confirmed cases of 21-day lag and formed 29 input features.

We calculated the Spearman correlation among 28 independent Google Trends volume data features in the 215 countries and territories to assess the independence of the features. We obtained the average results of the Spearman correlation coefficients between the top 10 cross-correlation Google volume data features in the 215 countries and territories. The highest correlation coefficient, between *coronavirus* interest over time and interest by region, was 0.59, which is lower than the criterion of strong correlation (0.8). Additionally, the cross-correlation between the remaining 18 Google volume data features was less than 0.1. Therefore, the correlation analysis results showed that the 28 Google volume data features were independent.

### Cluster Analysis

The internet search patterns and the management of COVID-19 vary among countries and territories. We used a hierarchical clustering technique to cluster the 215 countries and territories into several groups, and we built a model for each group. Two types of data were used for clustering. One type was the related metrics of the importance scores of 29 input features resulting from the random forest regression algorithm in 215 countries and territories. The other type was the correlation of the development trend of daily new confirmed cases in the 215 countries and territories. Finally, the 215 countries and territories were clustered into 8 groups (Multimedia Appendix 2). There were 150 countries and territories in the first group, 38 in the second group, 11 in the third group, 7 in the fourth group, 6 in the fifth group, and 1 country or territory each in the sixth, seventh, and eighth groups.

In addition, to explore the internal relationships between the countries and territories in each cluster, two types of Spearman correlation coefficients were calculated. The first was the Spearman correlation between the daily new confirmed cases among the countries in the first five groups, which contain more than one country or territory. The second was the Spearman correlation between the search volume of the term *coronavirus* among the countries in the first five groups. These two correlations were also calculated in the sixth, seventh, and eighth groups with a single country or territory. The average Spearman correlation coefficients are shown in Table 1. There were two findings. The first was a correlation between the incidence of COVID-19 among the countries and territories in each group, especially in the first group, which contains 150 countries and territories. The second was that the correlation of Google search data among countries and territories in the first five groups was high. These findings also showed the reliability of our clustering method.

**Table 1:** Average Spearman correlation coefficients in each group of countries/territories for the 2 data sets.

Data set		Average Spearman correlation coefficient							
		Group 1 (n=150)	Group 2 (n=38)	Group 3 (n=11)	Group 4 (n=7)	Group 5 (n=6)	Group 6 (n=1)	Group 7 (n=1)	Group 8 (n=1)
Daily new cases		0.46	0.18	0.35	0.26	0.38	0.23	0.04	0.15
*Coronavirus* search volume		0.76	0.68	0.71	0.74	0.69	0.52	0.00	0.43

### Modeling and Evaluation

We built a model for each group of countries/territories separately according to the clustering results and produced a 7-day-ahead incidence prediction of COVID-19 for the 215 countries and territories. The random forest regression algorithm has many decision trees; therefore, it has good robustness and a strong ability to resist overfitting. In addition, the random forest regression (RFR) algorithm gave the importance score of the features, which was helpful for feature selection. Therefore, the RFR algorithm was used to forecast the daily new confirmed cases of COVID-19 over the time series data set, which consisted of Google Trends data and the incidence of COVID-19 with 21-day lag provided by the WHO. Python 3.7.6 was used for the modeling and evaluation.

To quantitatively evaluate the performance of the models, we calculated four metrics: the mean absolute error (MAE), root mean square error (RMSE), Pearson correlation coefficient, and Spearman correlation coefficient.


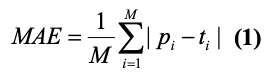



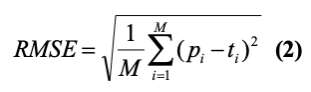



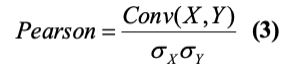



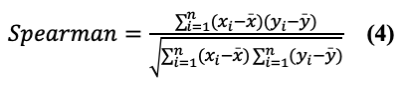


where *p_i_*, *t_i_*, and *M* represent the predicted value, true value, and total number of days; *X* and *Y* are two independent time series; and *σ_X_* and *σ_Y_* are the standard deviations of *X* and *Y*, respectively. *x_i_* and *y_i_* are the *i*th value of *X* and *Y* and 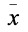 and 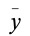 are the mean values of *X* and *Y*, respectively, and *n* is the length of the time series. The *P* values of the Pearson correlations were calculated to assess the statistical significance.

### Optimization and Validation

**Figure 2:**
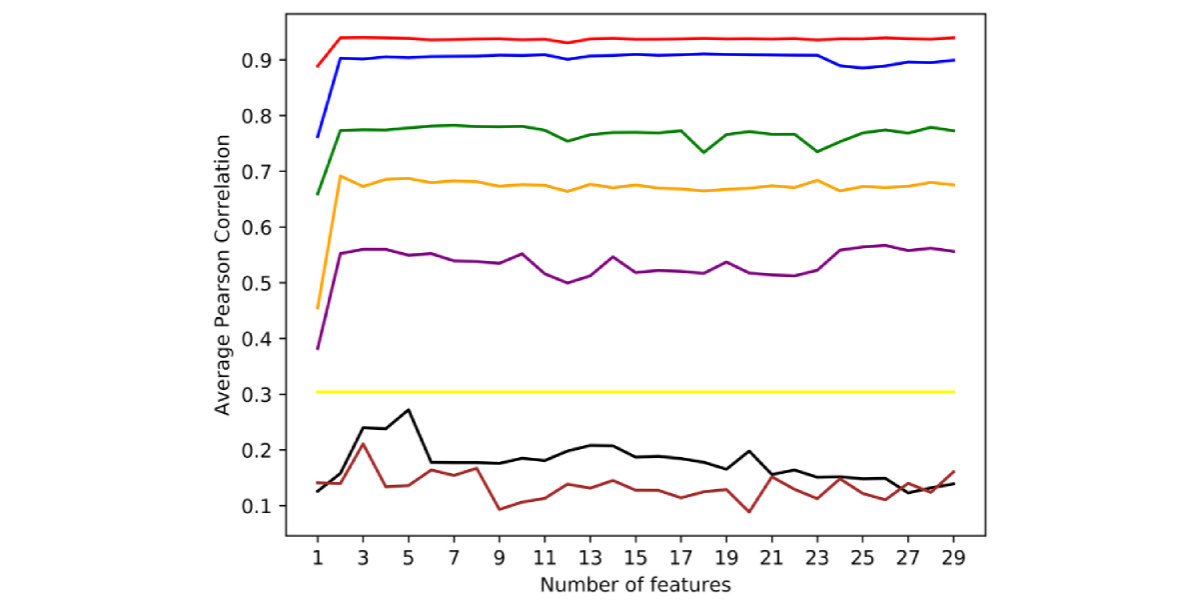
The average Pearson correlation coefficients of the number of top-scoring features included in the prediction of the incidence of COVID-19 in different groups of countries. The red, blue, green, orange, purple, yellow, black, and brown lines represent groups 1 to 8, respectively.

Several experiments were conducted to optimize and validate the results. First, to evaluate the role of each input feature in the prediction, we calculated the importance score of each input feature. To validate the features included in this study, a series of ablation studies, in which the top n features (n=1, 2, ..., 29) were used as inputs in turn, were conducted on different groups of countries/territories according to the clustering results. The Pearson correlation coefficient was averaged for the 8 groups of countries/territories based on the sum of the input features (Figure 2). According to the results, the top 13, 10, 10, 25, 2, 5, 1, and 3 features were used for the respective 8 groups in further experiments. The models using previous daily incidence with and without Google Trends data as input features were compared (Table 2). Adding Google search volume features as input improved the MAE, RMSE, Pearson correlation, and Spearman correlation by 0.28, 0.88, 0.06, and 0.08, respectively, in terms of average results in 215 countries and territories. These results confirmed that the Google Trends data played a positive role in predicting the incidence of COVID-19.

Second, to reduce the influence of random noise, we used multiple-step forecasting (5-step, 6-step, 7-step, 8-step, 9-step, and 10-step), which used the data over the past n days (n=5, 6, 7, 8, 9, 10) to predict the daily new confirmed cases of COVID-19. The average quantitative results of the different steps of the second period in the 215 countries and territories are shown in Table 3. The performance of the 7-step prediction was slightly better than that obtained using other numbers of steps in terms of 4 of 5 quantitative metrics in our study. Therefore, we selected the 7-step prediction.

Third, the proposed RFR algorithm was compared with two other mainstream algorithms. One was decision tree regression (DTR), which is a traditional machine learning algorithm. The other was long short-term memory (LSTM), which is a deep learning algorithm. Especially, LSTM, an artificial recurrent neural network, was used as a 3-layer model in our study. TensorFlow and Keras were used as frameworks for training the LSTM models. Table 4 shows the average quantitative results of the different methods for the 215 countries and territories. The random forest regression algorithm achieved better performance in terms of all quantitative metrics compared to DTR and LSTM.

Fourth, we trained the models with as much data as possible. Therefore, we updated the models and repeated the experiments at regular intervals. The effectiveness of the models at 3 time periods was compared.

Fifth, we predicted the incidence of COVID-19 2 days and 7 days ahead. Table 5 shows the average quantitative results of different time windows of the second period in the 215 countries and territories. We found the predictive power of the 7-day window to be less accurate than that of the 2-day window. It was noted that the 2-day window prediction may not be sufficiently informative for policy making, and the 7-day window would be more useful to enable a responsible authority to respond proactively and impose interventions in time. Therefore, we used the 7-day window for the prediction of the incidence of COVID-19.

**Table 2:** Average results without and with Google Trends data in 215 countries.

Method	MAE^a^				RMSE^b^				Pearson correlation				Spearman correlation				*P* value^c^			
	Avg^d^	Max^e^	Min^f^	Avg		Max	Min	Avg		Max	Min	Avg		Max	Min	Avg		Max	Min	
Without Google Trends	5.67	17.20	0.77	10.27		26.00	2.86	0.82		0.99	–0.02	0.76		0.99	–0.02	.018		<.999	<.001	
With Google Trends	5.42	15.32	0.26	9.27		24.40	1.81	0.89		0.99	0.08	0.84		1.00	0.21	.001		.24	<.001	

^a^MAE: mean absolute error.

^b^RMSE: root mean square error.

^c^*P* value of the Pearson correlation.

^d^Avg: average.

^e^Max: maximum.

^f^Min: minimum.

**Table 3:** The results of multiple-step forecasting in 215 countries and territories in the second period.

Method	MAE^a^	RMSE^b^	Pearson correlation	Spearman correlation	*P* value^c^
Step_5	5.48	9.35	0.88	0.84	.006
Step_6	5.46	9.21	0.88	0.84	.006
Step_7	5.44	9.12	0.88	0.84	.006
Step_8	5.66	9.49	0.88	0.84	.006
Step_9	5.66	9.46	0.88	0.85	.006
Step_10	5.70	9.50	0.88	0.84	.006

^a^MAE: mean absolute error.

^b^RMSE: root mean square error.

^c^*P* value of the Pearson correlation.

**Table 4:** Average performance of different methods in 215 countries and territories.

Method	MAE^a^	RMSE^b^	Pearson correlation	Spearman correlation	*P* value^c^
Decision tree regression	6.78	11.57	0.81	0.79	.011
Long short-term memory	9.13	14.29	0.76	0.78	.025
Random forest regression	5.42	9.27	0.89	0.84	.006

^a^MAE: mean absolute error.

^b^RMSE: root mean square error.

^c^*P* value of the Pearson correlation.

**Table 5:** The average results of different time windows of the second period in 215 countries and territories.

Time window	MAE^a^	RMSE^b^	Pearson correlation	Spearman correlation	*P* value^c^
2 days ahead	4.09	7.40	0.94	0.87	.003
7 days ahead	5.42	9.27	0.89	0.84	.006

^a^MAE: mean absolute error.

^b^RMSE: root mean square error.

^c^*P* value of the Pearson correlation.

## Results

**Figure 3:**
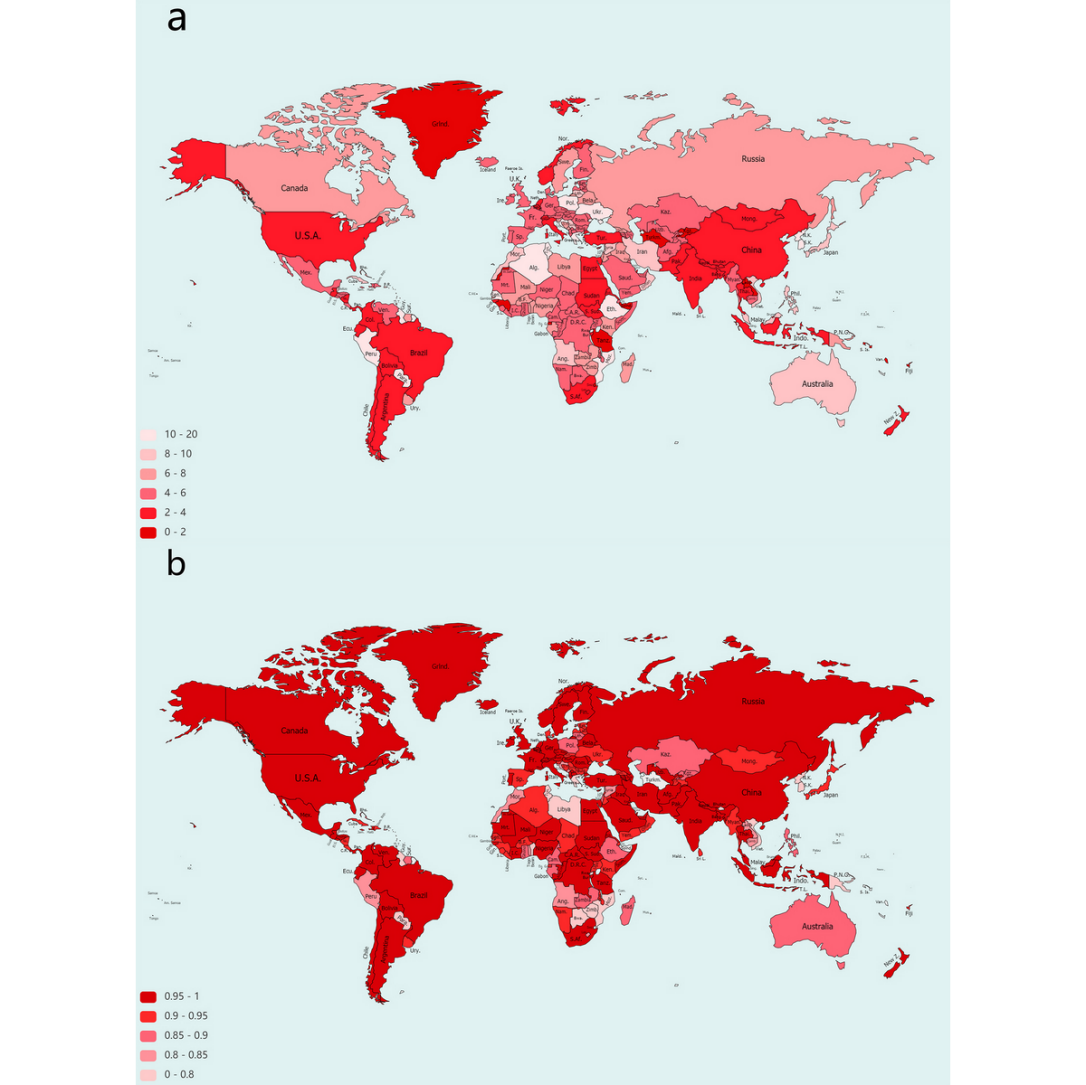
Heat maps of the (a) mean absolute error and (b) Pearson correlation coefficients of the predicted and actual daily new confirmed case numbers in different countries worldwide. The deeper the color, the lower the mean absolute error (a) and the higher the correlation coefficient (b).

We produced 7-day-ahead and real-time COVID-19 forecasts for 215 countries and territories. Our final models performed well in predicting the daily new confirmed cases of COVID-19 in most of the countries and territories examined. Of the 215 countries and territories, 198 (92.1%) had MAEs <10 (Figure 3a), and 187 (87.0%) had Pearson correlation coefficients >0.8 (Figure 3b). In all 215 countries/territories, the mean MAE was 5.42 (range 0.26-15.32), the mean RMSE was 9.27 (range 1.81-24.40), the mean Pearson correlation coefficient 0.89 (range 0.08-0.99) and mean Spearman correlation coefficient 0.84 (range 0.21-1.00), with *P*<.001 in most of the countries and territories. Detailed results of the individual countries and territories are listed in Multimedia Appendix 3. The average results for each group are shown in Table 6. Generally, the models performed well in the first, second, third, and fifth groups, which contained 150, 38, 11, and 6 countries and territories, respectively. The Pearson correlation coefficients were >0.80 in most of these countries and territories. In contrast, the performance was poorer in groups 4, 6, 7, and 8. The Pearson correlation coefficients were <0.80 in most of these countries.

**Figure 4:**
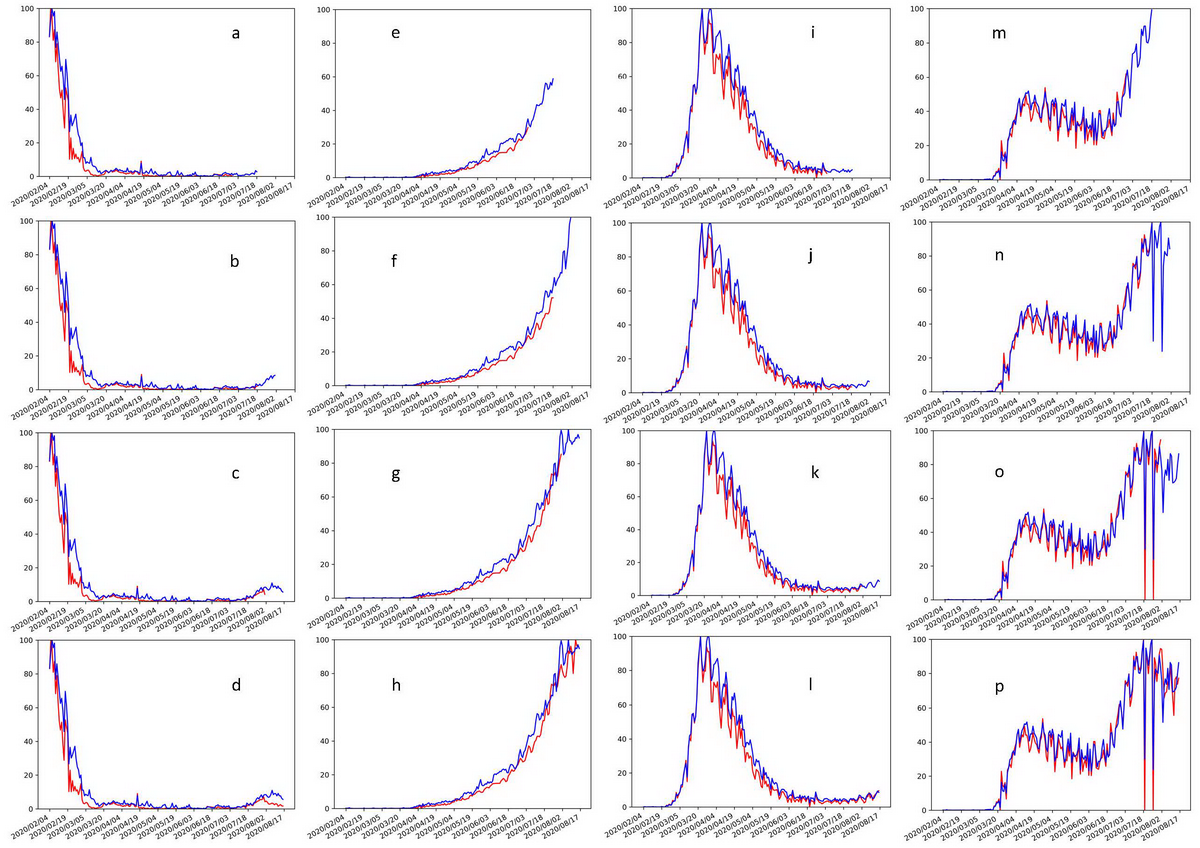
The prediction curves in China (a-d), India (e-h), Italy (i-l), and the United States (m-p). The blue and red lines indicate the predicted and real daily new cases, respectively. The first panels in each country (a, e, i, m) show the prediction for July 3 to July 17. The second panels (b, f, j, n) show the actual number of daily new cases for July 3 to July 17 and the prediction for July 18 to July 31. The third panels (c, g, k, o) show the actual number of daily new cases for July 18 to July 31 and the prediction for August 1 to August 16. The fourth panels (d, h, l, p) show the actual number of daily new cases from August 1 to August 16.

The performance of the models in 3 different time periods is shown in Table 7. As the training data increased, the overall prediction performance of the model improved. In addition, we randomly selected 4 countries from the 150 countries in the first group for further analysis. The predicted and actual daily new confirmed cases of the 3 different time periods in these 4 countries are shown in Figure 4. For example, our models can predict the increase in daily new confirmed cases in China from July 18 to July 31 and the decrease of daily new confirmed cases in China from August 3 to August 16. Our models were able to predict the daily new confirmed case numbers consistently.

**Table 6:** Performance of the final models in the different groups of countries/territories.

Cluster	Countries/ territories, n	MAE^a^			RMSE^b^				Pearson correlation				Spearman correlation				*P* value^c^		
		Avg^d^	Max^e^	Min^f^	Avg	Max	Min	Avg		Max	Min	Avg		Max	Min	Avg		Max	Min
1	150	5.52	15.00	0.26	8.96	23.45	3.32	0.94		0.99	0.72	0.92		1.00	0.74	<.001		<.001	<.001
2	38	3.92	10.18	0.36	7.17	14.73	1.81	0.90		0.98	0.65	0.67		0.90	0.25	<.001		<.001	<.001
3	11	7.38	13.26	1.71	13.77	20.68	5.97	0.77		0.89	0.70	0.81		0.92	0.63	<.001		<.001	<.001
4	7	6.41	8.82	1.32	14.01	16.86	8.83	0.52		0.63	0.39	0.60		0.76	0.42	<.001		<.001	<.001
5	6	6.12	15.32	1.76	13.01	23.19	4.99	0.66		0.86	0.50	0.71		0.79	0.58	<.001		<.001	<.001
6	1	5.72	5.72	5.72	12.75	12.75	12.75	0.27		0.27	0.27	0.58		0.58	0.58	<.001		<.001	<.001
7	1	2.39	2.39	2.39	2.39	12.17	12.17	0.30		0.30	0.30	0.46		0.46	0.46	<.001		<.001	<.001
8	1	14.60	14.60	14.60	24.40	24.40	24.40	0.008		0.008	0.008	0.21		0.21	0.21	.24		.24	.24
Total	215	5.42	15.32	0.26	9.27	24.40	1.81	0.89		0.99	0.08	0.84		1.00	0.21	.001		.24	<.001

^a^MAE: mean absolute error.

^b^RMSE: root mean square error.

^c^*P* value of the Pearson correlation.

^d^Avg: average.

^e^Max: maximum.

^f^Min: minimum.

**Table 7:** Performance of the models in different time periods.

Time period (2020)	MAE^a^				RMSE^b^				Pearson correlation				Spearman correlation				*P* value^c^		
	Avg^d^	Max^e^	Min^f^	Avg		Max	Min	Avg		Max	Min	Avg		Max	Min	Avg		Max	Min
Feb 04-July 17	10.11	20.88	0.31	15.76		33.15	3.94	0.79		0.99	-0.03	0.75		1.00	0.04	.009		<.999	<.001
Feb 04-July 31	5.44	15.98	0.28	9.12		25.02	2.71	0.88		0.99	-0.05	0.84		1.00	-0.13	.006		.83	<.001
Feb 04-Aug 16	5.42	15.32	0.26	9.27		24.20	1.81	0.89		0.99	0.08	0.84		1.00	0.21	.001		.24	<.001

^a^MAE: mean absolute error.

^b^RMSE: root mean square error.

^c^*P* value of the Pearson correlation.

^d^Avg: average.

^e^Max: maximum.

^f^Min: minimum.

## Discussion

### Principal Results

In this study, we have established a method to obtain a 7-day-ahead prediction of COVID-19 activity by combining the previous incidence of COVID-19 and Google search volume data at the country or territory level in the real world. The models performed well in most countries. In a total of 215 countries and territories, the mean MAE, RMSE, Pearson correlation and Spearman correlation values were 5.42, 9.27, 0.89, and 0.878, respectively. The *P* value was <.001 in most countries and territories, which shows the significance of the Pearson correlation coefficient. The results show that the longer the training time and the greater the amount of available data, the higher the prediction accuracy.

### Comparison With Prior Work

Our study has several advantages compared to other reported studies on similar topics. First, we investigated 215 individual countries and territories. Other studies only investigated a single country [15] or several countries [16], or they considered the world as one whole region [11,17]. Our method performed well in most of the countries and territories. Second, we used clustering methods to pool data with similar patterns together. The advantage of large amounts of data improved the performance of the models. Our results show that the groups with a large number of countries and territories had better results compared to those with a limited number of countries. There has been a report on clustering of provinces in China [12], but not clustering of countries. In another study, countries were grouped into 4 clusters; however, this clustering was not used for prediction [18]. Third, we combined data regarding both previous incidence of COVID-19 and Google Trends search volumes for prediction. Both of these data sources have been used individually in forecasting COVID-19 incidence. Our results showed that adding the Google Trends data improved the performance of the models developed from the previous incidence data. Fourth, we used machine learning methods for modeling. Many previous studies [13,19,20] indicated that Google Trends data are correlated with the incidence of COVID-19. Machine learning has been used to manage some large data sets and develop models to predict the incidence of COVID-19 in only a few countries [20]. Our models can produce useful and reliable predictions in most countries. Some studies used epidemical models such as susceptible-infected-recovered-dead (SIRD) for prediction. However, the spread of COVID-19 is affected by many factors, including public health management, traveling, and other social activities, which are not taken into account in the SIRD model. In Iran, COVID-19 was expected to subside at the beginning of May 2020 according to the SIRD model. However, at the time of writing this manuscript, thousands of new cases were still being reported daily in Iran [21]. Fifth, our models used the most up-to-date data and were updated three times. As more data become available, the performance of the models can be further improved. Some models developed several months ago may have severe errors. For example, Tuli et al [9] predicted that the total number of new cases would reach 97% of the total expected cases on August 14, 2020, in the United States [9]; however, tens of thousands of new cases were reported daily in July and August.

The high accuracy of our models enables real-time forecasting of the short-term trends of COVID-19, not only during the outbreak but also during the recovery period and subsequent second or third epidemic in individual countries. The methods can also be adapted to predict the incidence of subregions. Real-time digital surveillance of COVID-19 is provided, which would save time and resources in data collection. As the COVID-19 pandemic is changing rapidly, digital health systems should provide an effective solution to address the challenges to public health and consequential socioeconomical complications with high efficiency. The epidemic of COVID-19 is likely to persist in the future, and it may even become a seasonal infectious disease like influenza. Therefore, accurate surveillance and prediction of its activity would help governments, health care providers and the general population to take appropriate actions to compact the disruptive effects of COVID-19 [5].

### Limitations

We recognize some limitations of the current study. First, the models did not predict well in some countries, which were clustered into independent groups and had limited periods of data for training. Second, the occurrence of COVID-19 in some countries would have increased the awareness of other countries, especially those with a close relationship, consequently resulting in a large Google search volume for terms related to COVID-19. Third, there is a limitation in applying our models in countries and territories where Google is not the mainstream search engine. Thus, predictions of COVID-19 incidence in regions with limited internet access or prohibited Google access may not be accurate. Fourth, COVID-19 and influenza share some common symptoms and even prevention methods. Therefore, the Google Trends data of these terms may not be able to differentiate COVID-19 and influenza. Further studies are needed to develop an algorithm to differentiate these two infectious diseases using Google Trends.

### Conclusions

In this study, we integrated the Google Trends data and previous daily incidence of 215 individual countries and territories using the techniques of features engineering, country clustering, and machine learning. We are able to achieve prediction 7 days ahead of time of the daily incidence of COVID-19 in real time in most countries and territories.

## Appendix

Multimedia Appendix 1 Spearman correlation coefficients of 28 Google volume data features with the incidence of COVID-19 at n (n=7, 14, 21, 28) days of lag in 215 countries.

Multimedia Appendix 2 List of countries and territories with different clustering results.

Multimedia Appendix 3 Performance of the predicting model in 215 individual countries and territories.

## Abbreviations

DTR: decision tree regression
LSTM: long short-term memory
MAE: mean absolute error
RFR: random forest regression
RMSE: root mean square error
SIRD: susceptible-infected-recovered-dead
WHO: World Health Organization
