# Supplementary material for: Real-time Prediction of the Daily Incidence of COVID-19 in 215 Countries and Territories Using Machine Learning: Model Development and Validation
Source: J Med Internet Res. 2021 Jun 14;23(6):e24285. doi: 10.2196/24285 (PMC8204940; doi:10.2196/24285)
Supplement: Multimedia Appendix 1 [file jmir_v23i6e24285_app1.docx]

Multimedia Appendix 1. Spearman correlation coefficients of 28 Google volume data features with the incidence of COVID-19 at N (N=7,14,21,28) days lag in 215 countries.

| Lag days | 7 days | | 14 days | | 21 days | | 28 days | |
| --- | --- | --- | --- | --- | --- | --- | --- | --- |
|  | AVG | MAX | AVG | MAX | AVG | MAX | AVG | MAX |
| Coronavirus | 0.49 | 0.89 | 0.53 | 0.91 | 0.56 | 0.93 | 0.54 | 0.96 |
| Covid-19 | 0.40 | 0.89 | 0.43 | 0.92 | 0.47 | 0.93 | 0.46 | 0.88 |
| Cough | 0.17 | 0.45 | 0.16 | 0.51 | 0.17 | 0.54 | 0.16 | 0.59 |
| Diarrhea | 0.12 | 0.75 | 0.12 | 0.74 | 0.12 | 0.71 | 0.11 | 0.68 |
| Fatigue | 0.08 | 0.51 | 0.08 | 0.52 | 0.09 | 0.55 | 0.09 | 0.58 |
| Fever | 0.13 | 0.66 | 0.13 | 0.69 | 0.14 | 0.70 | 0.14 | 0.69 |
| Nasal congestion | 0.07 | 0.51 | 0.08 | 0.46 | 0.08 | 0.40 | 0.08 | 0.43 |
| Pneumonia | 0.13 | 0.73 | 0.13 | 0.65 | 0.14 | 0.68 | 0.13 | 0.69 |
| Rhinorrhea | 0.08 | 0.57 | 0.08 | 0.61 | 0.09 | 0.60 | 0.09 | 0.67 |
| Hand washing | 0.05 | 0.36 | 0.05 | 0.46 | 0.07 | 0.58 | 0.06 | 0.62 |
| Hand sanitizer | 0.13 | 0.65 | 0.16 | 0.75 | 0.18 | 0.84 | 0.17 | 0.91 |
| Mask | 0.23 | 0.83 | 0.23 | 0.80 | 0.22 | 0.75 | 0.20 | 0.73 |
| Social distance | 0.11 | 0.78 | 0.11 | 0.84 | 0.10 | 0.82 | 0.09 | 0.72 |
| Social isolation | 0.04 | 0.54 | 0.04 | 0.64 | 0.04 | 0.69 | 0.04 | 0.67 |
| Coronavirus_RE | 0.30 | 0.67 | 0.35 | 0.66 | 0.40 | 0.66 | 0.36 | 0.69 |
| Covid-19_RE | 0.17 | 0.61 | 0.20 | 0.56 | 0.21 | 0.66 | 0.22 | 0.74 |
| Cough_RE | 0.12 | 0.31 | 0.12 | 0.33 | 0.12 | 0.31 | 0.11 | 0.34 |
| Diarrhea_RE | 0.10 | 0.45 | 0.07 | 0.44 | 0.08 | 0.41 | 0.08 | 0.36 |
| Fatigue_RE | 0.08 | 0.18 | 0.07 | 0.33 | 0.09 | 0.23 | 0.08 | 0.32 |
| Fever_RE | 0.13 | 0.54 | 0.13 | 0.55 | 0.13 | 0.55 | 0.12 | 0.52 |
| Nasal congestion_RE | 0.07 | 0.33 | 0.08 | 0.33 | 0.07 | 0.35 | 0.07 | 0.31 |
| Pneumonia_RE | 0.13 | 0.65 | 0.13 | 0.63 | 0.14 | 0.64 | 0.14 | 0.69 |
| Rhinorrhea_RE | 0.07 | 0.28 | 0.08 | 0.33 | 0.08 | 0.35 | 0.07 | 0.33 |
| Hand washing_RE | 0.04 | 0.30 | 0.04 | 0.29 | 0.04 | 0.30 | 0.04 | 0.28 |
| Hand sanitizer_RE | 0.10 | 0.49 | 0.12 | 0.43 | 0.13 | 0.48 | 0.12 | 0.51 |
| Mask_RE | 0.13 | 0.67 | 0.13 | 0.61 | 0.12 | 0.63 | 0.12 | 0.64 |
| Social distance_RE | 0.09 | 0.54 | 0.09 | 0.51 | 0.08 | 0.49 | 0.07 | 0.54 |
| Social isolation_RE | 0.04 | 0.42 | 0.04 | 0.45 | 0.03 | 0.41 | 0.03 | 0.41 |

AVE: average; MAX: maximum
