# Supplementary material for: Real-time Prediction of the Daily Incidence of COVID-19 in 215 Countries and Territories Using Machine Learning: Model Development and Validation
Source: J Med Internet Res. 2021 Jun 14;23(6):e24285. doi: 10.2196/24285 (PMC8204940; doi:10.2196/24285)
Supplement: Multimedia Appendix 2 [file jmir_v23i6e24285_app2.docx]

Multimedia Appendix 2. List of countries/territories with different clustering results.

| category | n | list of countries/territories |
| --- | --- | --- |
| 1 | 150 | Afghanistan, Albania, Algeria, Andorra, Anguilla, Argentina, Armenia, Australia, Austria, Azerbaijan, Bahrain, Bangladesh, Belarus, Belgium, Bolivia (Plurinational State of), Bonaire, Sint Eustatius and Saba, Bosnia and Herzegovina, Brazil, British Virgin Islands, Brunei Darussalam, Bulgaria, Burkina Faso, Cabo Verde, Canada, Chad, Chile, China, Colombia, Congo, Costa Rica, Coted Ivoire, Croatia, Cuba, Cyprus, Czechia, Democratic Republic of the Congo, Denmark, Djibouti, Dominica, Dominican Republic, Ecuador, Egypt, El Salvador, Estonia, Eswatini, Ethiopia, Falkland Islands (Malvinas), Fiji, Finland, France, French Guiana, Georgia, Germany, Ghana, Greece, Greenland, Grenada, Guam, Guatemala, Guinea, Haiti, Honduras, Hungary, Iceland, India, Indonesia, Iran (Islamic Republic of), Iraq, Ireland, Italy, Jamaica, Japan, Jordan, Kazakhstan, Kenya, Kosovo, Kuwait, Laos, Latvia, Liberia, Lithuania, Luxembourg, Madagascar, Malawi, Malaysia, Mali, Mauritania, Mayotte, Mexico, Montenegro, Montserrat, Morocco, Mozambique, Myanmar, Namibia, Nepal, Netherlands, New Zealand, Niger, Nigeria, North Macedonia, Northern Mariana Islands (Commonwealth of the), Norway, occupied Palestinian territory, Oman, Pakistan, Panama, Paraguay, Peru, Philippines, Poland, Portugal, Puerto Rico, Qatar, Republic of Korea, Republic of Moldova, Reunion, Romania, Russian Federation, Saint Kitts and Nevis, Saint Lucia, Saint Pierre and Miquelon, San Marino, Saudi Arabia, Senegal, Serbia, Sierra Leone, Singapore, Slovakia, Slovenia, Somalia, South Africa, Spain, Sri Lanka, Suriname, Sweden, Switzerland, Tajikistan, The United Kingdom, Togo, Tunisia, Turkey, Uganda, Ukraine, United Arab Emirates, United States of America, Uruguay, Uzbekistan, Venezuela (Bolivarian Republic of), Yemen |
| 2 | 38 | Antigua and Barbuda, Bahamas, Bermuda, Burundi, Cambodia, Cameroon, Cayman Islands, Central African Republic, Comoros, Equatorial Guinea, Eritrea, Gabon, Gibraltar, Guernsey, Guinea-Bissau, Isle of Man, Israel, Jersey, Lebanon, Lesotho, Liechtenstein, Maldives, Mauritius, Monaco, Mongolia, New Caledonia, Nicaragua, Saint Vincent and the Grenadines, Sao Tome and Principe, Seychelles, South Sudan, Sudan, Syrian Arab Republic, Thailand, Timor-Leste, United Republic of Tanzania, United States Virgin Islands, Zambia |
| 3 | 11 | Angola, Barbados, Benin, Bhutan, Curacao, Libya, Malta, Rwanda, Trinidad and Tobago, Viet Nam, Zimbabwe |
| 4 | 7 | Belize, Botswana, Gambia, Guadeloupe, Martinique, Papua New Guinea, Saint Barthelemy |
| 5 | 6 | Faroe Islands, French Polynesia, Guyana, Kyrgyzstan, Sint Maarten, Turks and Caicos Islands |
| 6 | 1 | Aruba |
| 7 | 1 | Holy See |
| 8 | 1 | Saint Martin |
