# Supplementary material for: Real-time Prediction of the Daily Incidence of COVID-19 in 215 Countries and Territories Using Machine Learning: Model Development and Validation
Source: J Med Internet Res. 2021 Jun 14;23(6):e24285. doi: 10.2196/24285 (PMC8204940; doi:10.2196/24285)
Supplement: Multimedia Appendix 3 [file jmir_v23i6e24285_app3.docx]

Multimedia Appendix 3. Performance of the predicting model in 215 individual countries/territories

| Country/regions | cases | category | MAE | RMSE | Pearson | Spearman | *P* |
| --- | --- | --- | --- | --- | --- | --- | --- |
| Afghanistan | 37 596 | 1 | 4.87 | 7.14 | 0.977 | 0.9307 | < .001 |
| Albania | 7 260 | 1 | 5.47 | 7.92 | 0.9769 | 0.9402 | < .001 |
| Algeria | 38 133 | 1 | 11.89 | 18.31 | 0.9058 | 0.8715 | < .001 |
| Andorra | 997 | 1 | 6.18 | 9.91 | 0.9227 | 0.8364 | < .001 |
| Angola | 1 879 | 3 | 9.21 | 16.2 | 0.8158 | 0.8595 | < .001 |
| Anguilla | 3 | 1 | 0.26 | 3.58 | 0.9482 | 1 | < .001 |
| Antigua and Barbuda | 93 | 2 | 2.26 | 5.26 | 0.8416 | 0.5442 | < .001 |
| Argentina | 282 437 | 1 | 2.99 | 5.18 | 0.9832 | 0.9758 | < .001 |
| Armenia | 41 663 | 1 | 4.54 | 7.31 | 0.98 | 0.9706 | < .001 |
| Aruba | 894 | 6 | 5.72 | 12.75 | 0.2722 | 0.5839 | 0.0001 |
| Australia | 23 035 | 1 | 8.88 | 14.87 | 0.8765 | 0.8876 | < .001 |
| Austria | 23 211 | 1 | 4.07 | 6.78 | 0.967 | 0.9663 | < .001 |
| Azerbaijan | 34 107 | 1 | 5.63 | 11.01 | 0.9598 | 0.9763 | < .001 |
| Bahamas | 1 119 | 2 | 6.24 | 12.78 | 0.8732 | 0.8013 | < .001 |
| Bahrain | 46 430 | 1 | 5.51 | 7.87 | 0.977 | 0.9706 | < .001 |
| Bangladesh | 274 525 | 1 | 2.86 | 6.18 | 0.986 | 0.9789 | < .001 |
| Barbados | 148 | 3 | 3.78 | 10.9 | 0.7629 | 0.7932 | < .001 |
| Belarus | 69 424 | 1 | 6.05 | 8.48 | 0.9661 | 0.9092 | < .001 |
| Belgium | 77 776 | 1 | 3.95 | 6.04 | 0.9832 | 0.9254 | < .001 |
| Belize | 388 | 4 | 8.82 | 16.64 | 0.4295 | 0.5796 | < .001 |
| Benin | 2 063 | 3 | 7.99 | 13.58 | 0.7928 | 0.6305 | < .001 |
| Bermuda | 159 | 2 | 2.93 | 4.57 | 0.9371 | 0.6645 | < .001 |
| Bhutan | 133 | 3 | 3.61 | 9.04 | 0.726 | 0.7525 | < .001 |
| Bolivia (Plurinational State of) | 97 950 | 1 | 2.58 | 5.26 | 0.9853 | 0.9813 | < .001 |
| Bonaire, Sint Eustatius and Saba | 13 | 1 | 2.31 | 11.88 | 0.8336 | 0.9419 | < .001 |
| Bosnia and Herzegovina | 15 802 | 1 | 7.42 | 12.66 | 0.9107 | 0.8688 | < .001 |
| Botswana | 1 214 | 4 | 5.69 | 12.18 | 0.5443 | 0.427 | < .001 |
| Brazil | 3 275 520 | 1 | 3.99 | 6.46 | 0.984 | 0.9876 | < .001 |
| British Virgin Islands | 9 | 1 | 1.79 | 9.47 | 0.9083 | 0.9335 | < .001 |
| Brunei Darussalam | 142 | 1 | 2.27 | 5.62 | 0.948 | 0.8247 | < .001 |
| Bulgaria | 14 333 | 1 | 5.35 | 8.85 | 0.9285 | 0.9254 | < .001 |
| Burkina Faso | 1 240 | 1 | 6.32 | 8.78 | 0.9199 | 0.8593 | < .001 |
| Burundi | 413 | 2 | 2.69 | 5.25 | 0.9625 | 0.7234 | < .001 |
| Cabo Verde | 3 163 | 1 | 6.5 | 9.98 | 0.9505 | 0.9434 | < .001 |
| Cambodia | 273 | 2 | 5.14 | 9.14 | 0.835 | 0.7007 | < .001 |
| Cameroon | 18 469 | 2 | 5.96 | 8.67 | 0.8955 | 0.6697 | < .001 |
| Canada | 121 652 | 1 | 6.28 | 8.26 | 0.9768 | 0.9742 | < .001 |
| Cayman Islands | 203 | 2 | 4.37 | 7.84 | 0.896 | 0.7462 | < .001 |
| Central African Republic | 4 652 | 2 | 4.01 | 6.33 | 0.9779 | 0.7844 | < .001 |
| Chad | 952 | 1 | 4.83 | 7.62 | 0.9404 | 0.7811 | < .001 |
| Chile | 383 902 | 1 | 3.05 | 4.27 | 0.9605 | 0.9514 | < .001 |
| China | 89 761 | 1 | 3.49 | 6.22 | 0.9733 | 0.8651 | < .001 |
| Colombia | 445 111 | 1 | 3.94 | 7.14 | 0.9768 | 0.9806 | < .001 |
| Comoros | 405 | 2 | 1.97 | 3.65 | 0.9629 | 0.6879 | < .001 |
| Congo | 3 831 | 1 | 4.49 | 7.07 | 0.9692 | 0.9476 | < .001 |
| Costa Rica | 26 931 | 1 | 5.6 | 10.6 | 0.9444 | 0.9142 | < .001 |
| Coted Ivoire | 16 993 | 1 | 5.64 | 8.32 | 0.9576 | 0.8843 | < .001 |
| Croatia | 6 420 | 1 | 14.36 | 20.71 | 0.816 | 0.9024 | < .001 |
| Cuba | 3 292 | 1 | 8.39 | 12.13 | 0.9176 | 0.9722 | < .001 |
| Curacao | 32 | 3 | 1.71 | 5.97 | 0.8936 | 0.9248 | < .001 |
| Cyprus | 1 332 | 1 | 5.67 | 9.43 | 0.9239 | 0.9051 | < .001 |
| Czechia | 19 891 | 1 | 5.61 | 7.87 | 0.9663 | 0.9741 | < .001 |
| Democratic Republic of the Congo | 9 637 | 1 | 5.24 | 8.17 | 0.9631 | 0.946 | < .001 |
| Denmark | 15 483 | 1 | 5.47 | 8.77 | 0.9358 | 0.9629 | < .001 |
| Djibouti | 5 367 | 1 | 5.38 | 8.22 | 0.9255 | 0.8305 | < .001 |
| Dominica | 18 | 1 | 0.75 | 4.05 | 0.9365 | 0.8932 | < .001 |
| Dominican Republic | 85 545 | 1 | 5.5 | 9.66 | 0.9437 | 0.9595 | < .001 |
| Ecuador | 100 688 | 1 | 3.34 | 4.41 | 0.9534 | 0.9084 | < .001 |
| Egypt | 96 336 | 1 | 3.17 | 5.15 | 0.9897 | 0.9832 | < .001 |
| El Salvador | 22 619 | 1 | 4.07 | 6.02 | 0.9865 | 0.9824 | < .001 |
| Equatorial Guinea | 4 821 | 2 | 1.56 | 3.08 | 0.946 | 0.2486 | < .001 |
| Eritrea | 285 | 2 | 3.78 | 6.23 | 0.9531 | 0.5831 | < .001 |
| Estonia | 2 184 | 1 | 5.13 | 7.76 | 0.9471 | 0.9022 | < .001 |
| Eswatini | 3 745 | 1 | 7.55 | 13.19 | 0.8485 | 0.9018 | < .001 |
| Ethiopia | 28 894 | 1 | 12.63 | 20.29 | 0.8893 | 0.9341 | < .001 |
| Falkland Islands (Malvinas) | 13 | 1 | 0.56 | 3.32 | 0.9342 | 0.8144 | < .001 |
| Faroe Islands | 370 | 5 | 7.62 | 15.24 | 0.6768 | 0.7441 | < .001 |
| Fiji | 28 | 1 | 1.47 | 5.46 | 0.9193 | 0.8824 | < .001 |
| Finland | 7 720 | 1 | 5.11 | 7.09 | 0.9735 | 0.9497 | < .001 |
| France | 202 118 | 1 | 4.48 | 6.92 | 0.9587 | 0.9436 | < .001 |
| French Guiana | 8 588 | 1 | 5.48 | 9.32 | 0.9546 | 0.8804 | < .001 |
| French Polynesia | 166 | 5 | 3.84 | 9.4 | 0.6215 | 0.5769 | < .001 |
| Gabon | 8 225 | 2 | 7.13 | 10.43 | 0.8643 | 0.6921 | < .001 |
| Gambia | 1 689 | 4 | 7.83 | 16.86 | 0.3913 | 0.4562 | < .001 |
| Georgia | 1 336 | 1 | 7.45 | 10.43 | 0.9051 | 0.9084 | < .001 |
| Germany | 223 453 | 1 | 4.44 | 7.05 | 0.9817 | 0.9645 | < .001 |
| Ghana | 42 210 | 1 | 5.95 | 10.76 | 0.8857 | 0.8525 | < .001 |
| Gibraltar | 206 | 2 | 3.11 | 5.14 | 0.9473 | 0.6831 | < .001 |
| Greece | 6 858 | 1 | 12.33 | 18.35 | 0.7948 | 0.8401 | < .001 |
| Greenland | 14 | 1 | 0.68 | 3.38 | 0.967 | 0.9999 | < .001 |
| Grenada | 24 | 1 | 1.28 | 4.77 | 0.9177 | 0.8604 | < .001 |
| Guadeloupe | 446 | 4 | 7.37 | 14.22 | 0.58 | 0.6945 | < .001 |
| Guam | 508 | 1 | 8.12 | 11.94 | 0.8179 | 0.8331 | < .001 |
| Guatemala | 62 313 | 1 | 3.33 | 5.61 | 0.9634 | 0.9635 | < .001 |
| Guernsey | 252 | 2 | 2.52 | 5.69 | 0.9658 | 0.808 | < .001 |
| Guinea | 8 343 | 1 | 7.19 | 10.59 | 0.9515 | 0.9257 | < .001 |
| Guinea-Bissau | 2 117 | 2 | 3.05 | 4.87 | 0.953 | 0.6761 | < .001 |
| Guyana | 649 | 5 | 15.32 | 23.19 | 0.4961 | 0.6572 | < .001 |
| Haiti | 7 831 | 1 | 4.04 | 6.35 | 0.9727 | 0.9438 | < .001 |
| Holy See | 12 | 7 | 2.39 | 12.17 | 0.304 | 0.4577 | < .001 |
| Honduras | 49 467 | 1 | 4.42 | 9 | 0.9615 | 0.9782 | < .001 |
| Hungary | 4 877 | 1 | 5.97 | 7.95 | 0.9638 | 0.9503 | < .001 |
| Iceland | 1 999 | 1 | 4.08 | 6.79 | 0.9584 | 0.8945 | < .001 |
| India | 2 589 682 | 1 | 2.78 | 4.34 | 0.9938 | 0.979 | < .001 |
| Indonesia | 137 468 | 1 | 3.98 | 5.61 | 0.9855 | 0.9867 | < .001 |
| Iran (Islamic Republic of) | 341 070 | 1 | 8.17 | 9.78 | 0.983 | 0.9622 | < .001 |
| Iraq | 172 583 | 1 | 7.05 | 12.26 | 0.9651 | 0.9683 | < .001 |
| Ireland | 27 191 | 1 | 4.01 | 6.79 | 0.9799 | 0.9761 | < .001 |
| Isle of Man | 336 | 2 | 2.27 | 4.82 | 0.9515 | 0.7841 | < .001 |
| Israel | 88 488 | 2 | 7.39 | 14.13 | 0.8648 | 0.748 | < .001 |
| Italy | 253 438 | 1 | 3.3 | 5.09 | 0.9937 | 0.9837 | < .001 |
| Jamaica | 1 082 | 1 | 4.07 | 6.31 | 0.9114 | 0.9191 | < .001 |
| Japan | 54 714 | 1 | 8.44 | 12.56 | 0.9013 | 0.9068 | < .001 |
| Jersey | 356 | 2 | 3.82 | 6.2 | 0.9308 | 0.7095 | < .001 |
| Jordan | 1 339 | 1 | 7.01 | 9.63 | 0.9187 | 0.9222 | < .001 |
| Kazakhstan | 119 781 | 1 | 5.61 | 8.68 | 0.8587 | 0.8577 | < .001 |
| Kenya | 29 849 | 1 | 7.34 | 14.34 | 0.9007 | 0.9379 | < .001 |
| Kosovo | 11 232 | 1 | 4.53 | 7.85 | 0.9813 | 0.9594 | < .001 |
| Kuwait | 75 697 | 1 | 4.2 | 6.44 | 0.985 | 0.9774 | < .001 |
| Kyrgyzstan | 41 856 | 5 | 1.76 | 4.99 | 0.855 | 0.7863 | < .001 |
| Laos | 22 | 1 | 1.71 | 8.61 | 0.8399 | 0.8687 | < .001 |
| Latvia | 1 315 | 1 | 5.09 | 7.22 | 0.9445 | 0.9109 | < .001 |
| Lebanon | 8 442 | 2 | 9.09 | 12.61 | 0.9182 | 0.8127 | < .001 |
| Lesotho | 903 | 2 | 3.22 | 8.56 | 0.8474 | 0.624 | < .001 |
| Liberia | 1 257 | 1 | 4.89 | 7.23 | 0.9132 | 0.8843 | < .001 |
| Libya | 7 327 | 3 | 7.46 | 13.89 | 0.7638 | 0.7728 | < .001 |
| Liechtenstein | 93 | 2 | 1.55 | 3.61 | 0.9546 | 0.7877 | < .001 |
| Lithuania | 2 386 | 1 | 8.64 | 12.79 | 0.86 | 0.7979 | < .001 |
| Luxembourg | 7 439 | 1 | 5.62 | 9.51 | 0.9377 | 0.9173 | < .001 |
| Madagascar | 13 724 | 1 | 7.56 | 14.08 | 0.8975 | 0.924 | < .001 |
| Malawi | 5 026 | 1 | 4.66 | 9.05 | 0.9353 | 0.9101 | < .001 |
| Malaysia | 9 175 | 1 | 8.2 | 11.45 | 0.9692 | 0.9522 | < .001 |
| Maldives | 5 679 | 2 | 10.18 | 14.73 | 0.8374 | 0.9039 | < .001 |
| Mali | 2 614 | 1 | 7.05 | 9.78 | 0.9562 | 0.942 | < .001 |
| Malta | 1 247 | 3 | 11.13 | 17.03 | 0.7582 | 0.8241 | < .001 |
| Martinique | 336 | 4 | 6.51 | 13.48 | 0.5051 | 0.731 | < .001 |
| Mauritania | 6 693 | 1 | 4.14 | 6.96 | 0.9692 | 0.84 | < .001 |
| Mauritius | 346 | 2 | 2.34 | 5.06 | 0.948 | 0.688 | < .001 |
| Mayotte | 3 119 | 1 | 6.47 | 9.13 | 0.9331 | 0.8598 | < .001 |
| Mexico | 511 369 | 1 | 4.97 | 8 | 0.9804 | 0.9764 | < .001 |
| Monaco | 143 | 2 | 1.3 | 5.59 | 0.8482 | 0.478 | < .001 |
| Mongolia | 298 | 2 | 2.33 | 3.78 | 0.9413 | 0.6092 | < .001 |
| Montenegro | 4 013 | 1 | 6.51 | 11.71 | 0.8339 | 0.7455 | < .001 |
| Montserrat | 13 | 1 | 0.94 | 4.92 | 0.9007 | 0.8164 | < .001 |
| Morocco | 41 017 | 1 | 8.18 | 12.35 | 0.8367 | 0.8414 | < .001 |
| Mozambique | 2 791 | 1 | 15 | 23.45 | 0.7808 | 0.8826 | < .001 |
| Myanmar | 374 | 1 | 5.5 | 7.65 | 0.922 | 0.8077 | < .001 |
| Namibia | 3 907 | 1 | 4.91 | 9.58 | 0.9326 | 0.7751 | < .001 |
| Nepal | 26 019 | 1 | 3.1 | 6.59 | 0.9644 | 0.9437 | < .001 |
| Netherlands | 62 437 | 1 | 3.82 | 5.63 | 0.9838 | 0.984 | < .001 |
| New Caledonia | 23 | 2 | 2.95 | 11.46 | 0.6542 | 0.6705 | < .001 |
| New Zealand | 1 271 | 1 | 3.33 | 5.92 | 0.9731 | 0.8573 | < .001 |
| Nicaragua | 3 413 | 2 | 3.02 | 5.3 | 0.9487 | 0.2973 | < .001 |
| Niger | 1 165 | 1 | 5.65 | 8.99 | 0.976 | 0.9608 | < .001 |
| Nigeria | 48 770 | 1 | 6.34 | 10.24 | 0.9768 | 0.959 | < .001 |
| North Macedonia | 12 546 | 1 | 4.86 | 8.43 | 0.9614 | 0.9609 | < .001 |
| Northern Mariana Islands (Commonwealth of the) | 50 | 1 | 3.25 | 8.65 | 0.8685 | 0.8555 | < .001 |
| Norway | 9 850 | 1 | 3.51 | 5.52 | 0.969 | 0.9269 | < .001 |
| occupied Palestinian territory | 21 554 | 1 | 5.67 | 9.67 | 0.9579 | 0.8066 | < .001 |
| Oman | 82 924 | 1 | 8.77 | 13.97 | 0.9165 | 0.8857 | < .001 |
| Pakistan | 288 717 | 1 | 3.07 | 4.65 | 0.9867 | 0.9821 | < .001 |
| Panama | 79 402 | 1 | 2.87 | 4.43 | 0.9915 | 0.9835 | < .001 |
| Papua New Guinea | 275 | 4 | 7.36 | 15.85 | 0.6323 | 0.5738 | < .001 |
| Paraguay | 9 022 | 1 | 10.18 | 16.07 | 0.7771 | 0.8274 | < .001 |
| Peru | 516 296 | 1 | 13.71 | 20.59 | 0.8238 | 0.8762 | < .001 |
| Philippines | 157 918 | 1 | 8.05 | 12.04 | 0.8639 | 0.8878 | < .001 |
| Poland | 56 090 | 1 | 14.1 | 19.68 | 0.873 | 0.8206 | < .001 |
| Portugal | 53 981 | 1 | 5.21 | 6.77 | 0.9741 | 0.9644 | < .001 |
| Puerto Rico | 25 695 | 1 | 9.04 | 13.53 | 0.8433 | 0.8746 | < .001 |
| Qatar | 114 809 | 1 | 3.56 | 5.04 | 0.9897 | 0.9849 | < .001 |
| Republic of Korea | 15 318 | 1 | 10.6 | 16.02 | 0.7224 | 0.7405 | < .001 |
| Republic of Moldova | 29 905 | 1 | 4.3 | 6.46 | 0.9802 | 0.9832 | < .001 |
| Reunion | 788 | 1 | 2.97 | 5.57 | 0.8913 | 0.8376 | < .001 |
| Romania | 69 374 | 1 | 4.58 | 6.82 | 0.9763 | 0.9589 | < .001 |
| Russian Federation | 922 853 | 1 | 6.9 | 9.26 | 0.9835 | 0.961 | < .001 |
| Rwanda | 2 352 | 3 | 13.26 | 20.68 | 0.7274 | 0.9219 | < .001 |
| Saint Barthelemy | 13 | 4 | 1.32 | 8.83 | 0.5349 | 0.7612 | < .001 |
| Saint Kitts and Nevis | 17 | 1 | 0.98 | 4.18 | 0.913 | 0.8503 | < .001 |
| Saint Lucia | 25 | 1 | 1.3 | 5.27 | 0.9346 | 0.8974 | < .001 |
| Saint Martin | 109 | 8 | 14.6 | 24.4 | 0.0847 | 0.2109 | 0.2391 |
| Saint Pierre and Miquelon | 4 | 1 | 0.51 | 5.06 | 0.942 | 1 | < .001 |
| Saint Vincent and the Grenadines | 57 | 2 | 2.07 | 5.04 | 0.898 | 0.646 | < .001 |
| San Marino | 719 | 1 | 4.73 | 7.2 | 0.9588 | 0.8144 | < .001 |
| Sao Tome and Principe | 885 | 2 | 2.09 | 3.97 | 0.9382 | 0.5512 | < .001 |
| Saudi Arabia | 297 315 | 1 | 4.7 | 6.62 | 0.9859 | 0.9865 | < .001 |
| Senegal | 12 032 | 1 | 12.14 | 15.8 | 0.9469 | 0.9527 | < .001 |
| Serbia | 29 471 | 1 | 8.28 | 12.35 | 0.9311 | 0.9402 | < .001 |
| Seychelles | 127 | 2 | 2.7 | 6.33 | 0.8035 | 0.5125 | < .001 |
| Sierra Leone | 1 954 | 1 | 4.14 | 6.15 | 0.9479 | 0.9574 | < .001 |
| Singapore | 55 661 | 1 | 9.05 | 12.77 | 0.9373 | 0.9506 | < .001 |
| Sint Maarten | 263 | 5 | 7 | 13.97 | 0.6584 | 0.7123 | < .001 |
| Slovakia | 2 855 | 1 | 5.94 | 8.81 | 0.9274 | 0.937 | < .001 |
| Slovenia | 2 401 | 1 | 7.57 | 10.69 | 0.9468 | 0.953 | < .001 |
| Somalia | 3 256 | 1 | 5.93 | 8.64 | 0.9649 | 0.8838 | < .001 |
| South Africa | 583 653 | 1 | 2.79 | 6.44 | 0.9822 | 0.9668 | < .001 |
| South Sudan | 2 489 | 2 | 2.81 | 4.76 | 0.9548 | 0.7749 | < .001 |
| Spain | 342 813 | 1 | 5.27 | 9.86 | 0.9353 | 0.9006 | < .001 |
| Sri Lanka | 2 886 | 1 | 4.3 | 5.79 | 0.9408 | 0.8498 | < .001 |
| Sudan | 12 314 | 2 | 4.66 | 8.35 | 0.8444 | 0.6711 | < .001 |
| Suriname | 2 838 | 1 | 7.06 | 13.24 | 0.8991 | 0.8861 | < .001 |
| Sweden | 84 294 | 1 | 6.19 | 7.85 | 0.9663 | 0.9357 | < .001 |
| Switzerland | 37 831 | 1 | 2.69 | 4.64 | 0.9792 | 0.9593 | < .001 |
| Syrian Arab Republic | 1 593 | 2 | 8.13 | 12.69 | 0.8323 | 0.7517 | < .001 |
| Tajikistan | 8 029 | 1 | 5.77 | 8.72 | 0.9492 | 0.9062 | < .001 |
| Thailand | 3 377 | 2 | 3.73 | 5.97 | 0.9592 | 0.6672 | < .001 |
| The United Kingdom | 316 371 | 1 | 4.68 | 6.56 | 0.9888 | 0.9787 | < .001 |
| Timor-Leste | 25 | 2 | 0.36 | 1.81 | 0.9768 | 0.7797 | < .001 |
| Togo | 1 092 | 1 | 7.68 | 13.41 | 0.843 | 0.8538 | < .001 |
| Trinidad and Tobago | 474 | 3 | 6.9 | 11.92 | 0.7962 | 0.784 | < .001 |
| Tunisia | 2 023 | 1 | 11.64 | 16.48 | 0.7965 | 0.9176 | < .001 |
| Turkey | 248 117 | 1 | 3.82 | 5.4 | 0.9876 | 0.974 | < .001 |
| Turks and Caicos Islands | 274 | 5 | 4.15 | 11.27 | 0.6743 | 0.7943 | < .001 |
| Uganda | 1 385 | 1 | 4.27 | 6.24 | 0.9243 | 0.8416 | < .001 |
| Ukraine | 91 356 | 1 | 12.99 | 17.73 | 0.9176 | 0.9472 | < .001 |
| United Arab Emirates | 64 102 | 1 | 4.19 | 6.05 | 0.9839 | 0.9742 | < .001 |
| United Republic of Tanzania | 509 | 2 | 1.65 | 2.91 | 0.9615 | 0.4122 | < .001 |
| United States of America | 5 258 565 | 1 | 3.07 | 5.3 | 0.9843 | 0.9785 | < .001 |
| United States Virgin Islands | 734 | 2 | 7.7 | 12.89 | 0.8373 | 0.7734 | < .001 |
| Uruguay | 1 421 | 1 | 6.21 | 8.92 | 0.9359 | 0.9475 | < .001 |
| Uzbekistan | 34 944 | 1 | 4.23 | 6.87 | 0.9746 | 0.9545 | < .001 |
| Venezuela (Bolivarian Republic of) | 31 381 | 1 | 5.18 | 8.54 | 0.9605 | 0.9231 | < .001 |
| Viet Nam | 951 | 3 | 8.39 | 14.63 | 0.7027 | 0.7273 | < .001 |
| Yemen | 1 862 | 1 | 5.56 | 8.64 | 0.9154 | 0.909 | < .001 |
| Zambia | 9 186 | 2 | 6.75 | 12.78 | 0.851 | 0.7466 | < .001 |
| Zimbabwe | 5 176 | 3 | 7.76 | 17.58 | 0.7665 | 0.8773 | < .001 |
